# Antimicrobial Resistance in *Pasteurella multocida* Isolates from Bovine Mastitis Can Be Associated with Multidrug-Resistance-Mediating Integrative and Conjugative Elements (ICEs)

**DOI:** 10.3390/antibiotics14020153

**Published:** 2025-02-04

**Authors:** Johanna Jahnen, Dennis Hanke, Kristina Kadlec, Stefan Schwarz, Henrike Krüger-Haker

**Affiliations:** 1 Institute of Microbiology and Epizootics, School of Veterinary Medicine, Freie Universität Berlin, 14163 Berlin, Germany; johanna.jahnen@fu-berlin.de (J.J.); dennis.hanke@fu-berlin.de (D.H.); henrike.krueger@fu-berlin.de (H.K.-H.); 2 Veterinary Centre for Resistance Research (TZR), School of Veterinary Medicine, Freie Universität Berlin, 14163 Berlin, Germany; 3 Dairy Herd Consulting and Research Company (MBFG), 31515 Wunstorf, Germany; kkadlec@gmx.de

**Keywords:** diagnostic sample, cattle, *Pasteurellaceae*, antimicrobial resistance surveillance, broth microdilution, whole-genome sequencing (WGS), mobile genetic elements

## Abstract

**Background/Objectives**: *Pasteurella multocida* commonly colonizes the bovine respiratory tract and can occasionally cause intramammary infections. Here, eight *P. multocida* isolates from clinical cases of bovine mastitis were investigated for their molecular characteristics as well as phenotypic and genotypic antimicrobial resistance (AMR) properties. **Methods**: The isolates originated from quarter milk samples obtained in Germany for diagnostic purposes. Antimicrobial susceptibility testing (AST) by broth microdilution was performed according to the Clinical and Laboratory Standards Institute. Closed whole-genome sequences were generated by hybrid assembly of Illumina MiSeq short-reads and Oxford Nanopore MinION long-reads, followed by consecutive sequence analysis. **Results**: The *P. multocida* isolates belonged either to capsular:lipopolysaccharide type A:3 (*n* = 7) or A:6 (*n* = 1), and multi-locus sequence types 1 (*n* = 7) or 7 (*n* = 1). Seven isolates carried AMR genes, such as *mef*(C), *mph*(G), *strA*, *strB*, *aphA1*, *aadA31*, *tet*(H), *tet*(Y), *floR*, *catA3*, and *sul2*, as part of an integrative and conjugative element (ICE). These mobile genetic elements, 58,382–78,401 bp in size, were highly similar to the ICEs Tn*7406* or Tn*7407* that have been previously described in bovine *Mannheimia haemolytica* and *P. multocida*, respectively. Moreover, the isolates showed elevated minimal inhibitory concentrations corresponding to the identified AMR determinants. **Conclusions**: Molecular typing and ICE organization suggest the bovine respiratory tract as reservoir of the investigated mastitis-associated *P. multocida*. Horizontal cross-genus transfer of multidrug-resistance-mediating ICEs seems to occur under in vivo conditions among different pathogens from cattle in Germany, which underlines the importance of pathogen identification followed by AST for successful bovine mastitis therapy.

## 1. Introduction

*Pasteurella multocida* is a Gram-negative, facultatively anaerobic, non-motile rod belonging to the genus *Pasteurella* within the family *Pasteurellaceae*. It is an opportunistic pathogen, whose natural reservoir is the upper respiratory tract of mammals and birds [1]. As a potentially zoonotic bacterium, *P. multocida* can be involved in a variety of diseases in different host species [1]. In humans, *P. multocida* infections are mainly associated with bites or scratches caused by cats and dogs, although non-bite transmissions also occur [2,3]. The most common associated disorders in cattle are bovine respiratory disease (BRD) and hemorrhagic septicemia; however, *P. multocida* has occasionally also been reported as the causative agent of bovine mastitis [1,4,5,6,7]. Mastitis caused by *Pasteurella* spp. has been shown to come along with large production losses [7]. In addition to cattle, *P. multocida* has been described to cause mastitis in rabbits [8], dairy camels [9,10], sheep [1], and a goat [11]. Mastitis is characterized by inflammation of the mammary gland, which is almost always caused by infecting microorganisms [12]. When udder inflammation is characterized by visible abnormalities in the milk and/or the udder, this is defined as clinical mastitis, whereas an increase only in the milk somatic cell count without clinical signs should be reported as subclinical mastitis [12]. *P. multocida*-associated cases of bovine mastitis may be subclinical to clinical with not only changes in the appearance of the milk and/or the udder but also systemic signs of infection [6]. Bacteria causing mastitis most often enter the mammary gland via the teat canal and cause inflammation in a single quarter of the udder of a cow. More likely, *P. multocida* spreads via cow–cow contact or during milking, but the bacteria entering the quarter may also originate from the environment of the cow. Lymphogenic or hematogenic spread of *Pasteurella* spp. from the respiratory tract to the udder has also been mentioned [13]. Moreover, several studies have discussed the transmission of *P. multocida* via suckling calves [6,14,15,16]. Köllmann et al. showed high similarities between *P. multocida* strains detected in the mammary glands of foster cows and those in the oral cavities of the respective associated foster calves, indicating that transmission of *P. multocida* via suckling calves appears very likely [16]. Notably, a study from Switzerland revealed *P. multocida* as the most common mastitis pathogen in suckler cows [14]. This observation was in contrast to studies focusing on dairy cows and the fact that Gram-negative mastitis pathogens have only rarely been reported from suckler cows [14]. Since calves from dairy cows are, unlike beef cattle, usually raised separately, potential pathogen transmission via suckling calves might explain why *P. multocida*-associated mastitis is observed less frequently in dairy cows [14]. Another previous publication has shown that the same *P. multocida* strain can cause respiratory disease as well as clinical mastitis in cattle; however, this connection was observed in the absence of active nursing of calves [4].

Mastitis is an ongoing problem worldwide, especially in the dairy industry, that is not only associated with reduced animal welfare and increased treatment costs but also loss in milk yield and quality, decreased fertility, as well as an increased risk of culling and death [17,18]. Consequently, the therapy of bovine mastitis is of high relevance for both economic considerations as well as animal health and welfare. In this regard, antimicrobial treatment is often indispensable [19]. The chosen approach is usually dependent on the existence and severity of clinical symptoms and the time of lactation in which the illness or variation in milk quality occurs [20]. For subclinical mastitis, it is often recommended to start focal (i.e., intramammary) treatment at the end of the lactation to ensure complete microbiological healing and prevent new infections during the dry period [21,22]. In contrast, for clinical cases of mastitis, immediate reaction is necessary both for the wellbeing of the cow and to ensure future milk production [22]. According to the German Veterinary Information Service for Drug Use, Toxicology, and Drug Law [23], antimicrobial agents for the treatment of bovine mastitis can be licensed in Germany either for the intramammary treatment of subclinical mastitis and clinical cases without fever, respectively, or systemic application. The licensed antimicrobial drugs in Germany for intramammary treatment include penicillins (amoxicillin/clavulanic acid, ampicillin, cloxacillin, nafcillin, oxacillin, penicillin, and penethamate), first- and fourth-generation cephalosporins (cefalexin, cefalonium, cefapirin, and cefquinome), aminoglycosides (kanamycin, neomycin, and streptomycin), and lincosamides (lincomycin and pirlimycin). Several combination preparations of these substances are available. Licensed pharmaceuticals for systemic application include penicillins (amoxicillin, amoxicillin/clavulanic acid, penicillin with or without neomycin, and penethamate), the fourth-generation cephalosporin cefquinome, a tetracycline (oxytetracycline), a macrolide (tylosin), and fluoroquinolones (danofloxacin, enrofloxacin, and marbofloxacin). The antimicrobial agent for treatment should be chosen based on the species of the causative pathogen and in accordance with an antibiogram. Depending on the severity of the symptoms, the causative microbial pathogen, and the available composition of the chosen antimicrobial agent, systemic administration may be preferred over an intramammary dispenser, and an anti-inflammatory drug, such as glucocorticoids, might be added [24]. It is worth noting that there is no preparation available in Germany that is licensed specifically for the treatment of bovine mastitis caused by *P. multocida*. In a study from Spain, cows infected with *P. multocida* were treated via intramammary infusion for two days with cefalexin and kanamycin, followed by intramammary dry infusion for one day of penethamate (the diethylaminoethyl ester of benzylpenicillin), penicillin, and framycetin (a component of neomycin) [4]. In addition, the macrolide tilmicosin was given parenterally, and eventually, the treatments resolved the infections [4]. A cow from a dairy farm in Wisconsin, the United States of America (USA), suffering from severe clinical mastitis due to *Pasteurella* spp. received systemic sulfadimethoxine, a sulfonamide [18]. A case report from Serbia described the empirical intramammary administration of a suspension of tetracycline, neomycin, bacitracin (a polypeptide antibiotic), and prednisolone, which proved to be inefficient [6]. After treatment was changed to the intramammary application of penicillin and dexamethasone for five consecutive days, the infection was resolved [6].

Antimicrobial resistance (AMR) in *P. multocida* is commonly associated with integrative and conjugative elements (ICEs) [1,25,26,27,28,29,30,31]. These self-transferable mobile genetic elements (MGEs) integrate site-specifically into the chromosomal DNA. During transmission, a circular intermediate is formed after excision of the ICE from the chromosomal DNA of the donor. This circular intermediate transfers conjugatively into another host bacterium and, there, eventually integrates into the chromosomal DNA [32]. Besides core genes essential for transfer and regulation, ICEs usually harbor accessory genes, including AMR genes [32]. Thus, ICEs seem to play a crucial role in the dissemination of AMR genes among members of the *Pasteurellaceae* family, which may act as donors and/or recipients [1]. Moreover, multidrug resistance (MDR), which means resistance to at least one agent in three or more antimicrobial classes [33], is increasingly reported among *P. multocida* associated with BRD and commonly mediated by ICEs [25,30,34,35]. The presence of multiple resistance genes located on the same ICE offers excellent opportunities for co-selection and persistence of these ICEs and ensures that such MDR-mediating ICEs are not easily lost. Few studies investigated the phenotypic AMR profiles of *P. multocida* isolates from mastitis [9,14]. Elevated minimal inhibitory concentrations (MICs) or decreased growth inhibition zones have been described, for example, for several β-lactams, pirlimycin, kanamycin, neomycin, or spiramycin [9,14]. However, the corresponding genotypic data were not reported [9,14].

Here, eight *P. multocida* isolates from clinical cases of bovine mastitis were investigated for their molecular features, as well as phenotypic and genotypic AMR properties. Applying whole-genome sequencing (WGS) and antimicrobial susceptibility testing (AST) by broth microdilution, the aim was to elucidate whether the isolates’ characteristics differed from those previously revealed for bovine respiratory tract pathogens belonging to the *Pasteurellaceae* family [1,25,26,34,36,37].

## 2. Results

### 2.1. Molecular Typing and Phylogenetic Analysis

The eight *P. multocida* isolates investigated in this study were detected in quarter milk samples from cows suffering from clinical mastitis. The individual characteristics of the isolates are displayed in Table 1. Seven isolates belonged to sequence type (ST) 1 (*adk*_1, *aroA*_1, *deoD*_1, *gdhA*_1, *g6pd*_1, *mdh*_1, *pgi*_1), while one isolate represented ST7 (*adk*_4, *aroA*_4, *deoD*_4, *gdhA*_1, *g6pd*_4, *mdh*_6, *pgi*_4). The ST1 isolates also showed the same capsular:lipopolysaccharide (CAP:LPS) type, A:3, whereas the ST7 isolate displayed CAP:LPS type A:6. Considering the CAP type, the capsule biosynthetic locus in isolates 1 and 6 to 8 matched allele *capA4*, while isolate 2 harbored allele *capA9*. The best match of isolates 3, 4, and 5, taking into account the alleles available in the database [38], was also allele *capA4*. Although the capsule biosynthetic locus here was organized as described for serogroup A by Townsend et al. [39], a mutation was detected at position A2696C of the *hyaD* gene, resulting in the amino acid exchange P→Q. Therefore, the CAP type of these isolates was not identifiable in the database via automatic query [38].

**Figure 1:**
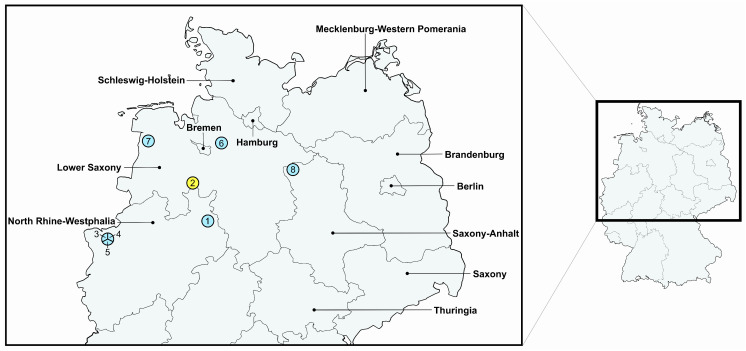
Distribution of the *P. multocida* isolates 1–8 from bovine mastitis included in this study within Germany based on zip code regions. The isolate IDs 1–8 are given within or outside the circles, respectively. Isolates 3, 4, and 5, which are associated with one circle, originated from the same farm. The colors of the circles refer to the different STs of the isolates: ST1 in blue and ST7 in yellow.

The quarter milk samples in which the *P. multocida* isolates were detected originated from farms in three neighboring federal states of Germany—North Rhine-Westphalia, Lower Saxony, and Saxony-Anhalt (Figure 1). Notably, isolates 3, 4, and 5 originated from the same organic farm in North Rhine-Westphalia. More precisely, isolates 3 and 4 were even obtained from the same animal but from different udder quarters. The complete genomes of the isolates ranged between 2,277,048 and 2,410,133 bp in size. They shared a core genome length of 1,773,184 bp with a total of 13,993 extracted single-nucleotide polymorphisms (SNPs). SNP comparisons revealed that isolate 2 differed most from the remaining seven isolates with 13,846 to 13,878 SNPs (Table 2). This isolate belonged to a different ST as well as CAP:LPS type and did not carry an ICE (Table 1). Comparing the seven ST1/A:3 isolates, isolate 8 showed the most differences ranging between 174 and 177 SNPs (Table 2). This isolate was the only one originating from Saxony-Anhalt and harbored a different kind of ICE (Figure 1, Table 1). The isolates 3, 4, and 5, which were found in samples from the same farm in 2022, displayed the closest relationships differing only in 2 to 13 SNPs (Table 2). Isolates 1, 6, and 7, which have been obtained from samples in North-Rhine Westphalia and Lower Saxony, respectively, differed from the other ST1/A:3 isolates rather similarly in 18 to 43 SNPs, thus still showing rather close relationships (Figure 1, Table 2).

### 2.2. Phenotypic AMR Profiles

The individual phenotypic AMR profiles of the isolates are displayed in Table 1. One isolate, ID 2, did not show any increased MIC values, whereas the remaining seven isolates displayed a phenotypic MDR profile. Elevated MIC values were observed for differing antimicrobial agents belonging to the groups of β-lactams, macrolides, aminoglycosides, tetracyclines, phenicols, and folate pathway inhibitors. However, clinical breakpoints for bovine *P. multocida* isolates from intramammary infections are not available to date [40]. Consequently, the limited breakpoints available from the Clinical and Laboratory Standards Institute (CLSI) for *P. multocida* isolates from bovine respiratory tract infections, other animal species, and humans, respectively, can only be used as an orientation in the MIC value interpretation (Table A1) [40,41].

### 2.3. Genotypic AMR Profiles

**Figure 2:**
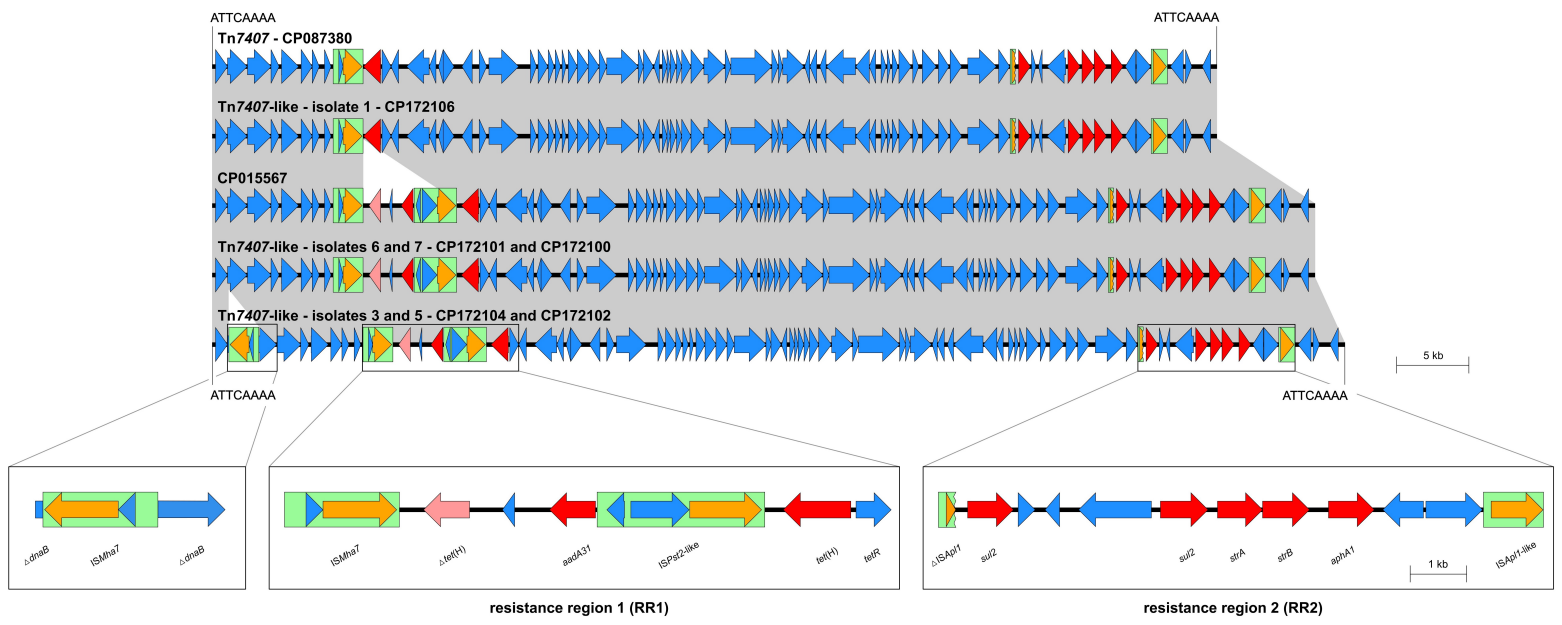
Organization of the Tn*7407*-like ICEs and their resistance gene regions found among five of the eight *P. multocida* isolates included in this study (GenBank accession nos. CP172106, CP172101, CP172100, CP172104, and CP172102) aligned to the sequences of ICE Tn*7407* (GenBank accession no. CP087380) and that of a bovine *P. multocida* from GenBank (accession no. CP015567). Open reading frames are shown as arrows, with the arrowhead indicating the direction of transcription. Resistance genes are marked in red, truncated resistance genes in light red, transposase genes in orange, and other genes and genes with unknown functions in blue. IS elements are displayed as green boxes, and homologous regions between sequences are indicated by gray shading. Size scales are given on the right-hand side.

**Figure 3:**
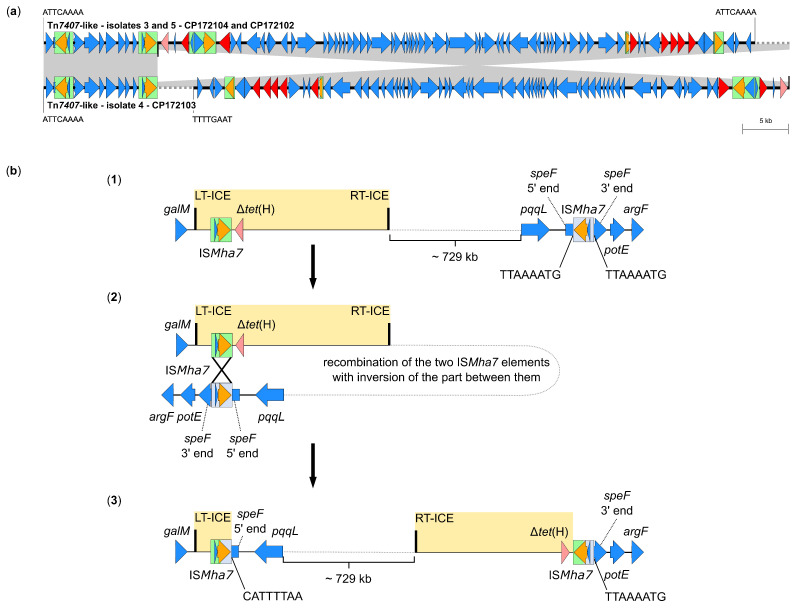
A disrupted Tn*7407*-like ICE with a segment of circa 732,885 bp located between its left-hand and right-hand parts was found in one of the eight *P. multocida* isolates included in this study. (**a**) Organization of the disrupted ICE (GenBank accession no. CP172103) aligned to the sequence of the Tn*7407*-like ICE detected in two isolates from the same farm (GenBank accession nos. CP172104 and CP172102). Open reading frames are shown as arrows, with the arrowhead indicating the direction of transcription. Resistance genes are marked in red, truncated resistance genes in light red, transposase genes in orange, and other genes and genes with unknown functions in blue. IS elements are displayed as green boxes, and homologous regions between sequences are indicated by gray shading; the right-hand part of the disrupted ICE is present in the reverse complementary orientation. A size scale is given on the right-hand side. (**b**) Possible development of the ICE disruption in isolate 4: (**1**) shows the hypothetical situation prior to the genetic rearrangement, which notably was observed in isolate 5 from the same farm carrying the complete variant of this ICE. An IS*Mha7* element was integrated into the *speF* gene. In the following, a homologous recombination of the two IS*Mha7* elements in reverse orientation with inversion of the part between them (**2**) might have resulted in the separation of the left- and right-hand ICE parts, as displayed in (**3**).

**Figure 4:**
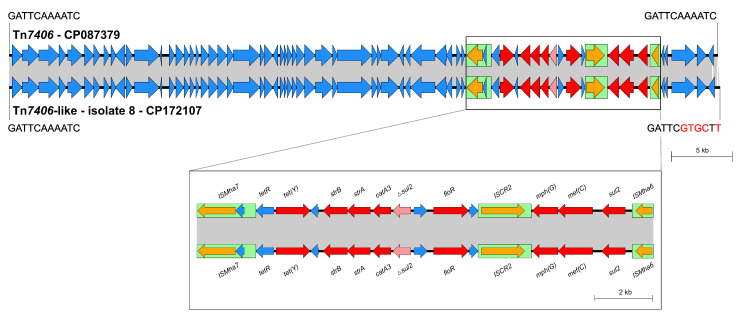
Organization of the Tn*7406*-like ICE and its resistance gene region found among one of the eight *P. multocida* isolates included in this study (GenBank accession no. CP172107) aligned to the sequence of ICE Tn*7406* (GenBank accession no. CP087379). Open reading frames are shown as arrows, with the arrowhead indicating the direction of transcription. Resistance genes are marked in red, truncated resistance genes in light red, transposase genes in orange, and other genes and genes with unknown functions in blue. IS elements are displayed as green boxes, and homologous regions between sequences are indicated by gray shading. Size scales are given on the right-hand side.

The individual AMR genotypes of the isolates can also be found in Table 1. Overall, the phenotypic AMR profiles of the isolates correlated almost completely with the respective resistance genotypes. In line with the non-elevated MIC values, isolate 2 did not harbor any known genotypic AMR determinants. In isolates 1 and 3 to 8, which showed elevated MICs of β-lactams, no explanatory genotypic determinant could be identified for this observation. Common β-lactamase genes, such as *bla*_CMY-2_, *bla*_OXA-2_, *bla*_PSE-1_, *bla*_ROB-1_, *bla*_ROB-13_, or *bla*_TEM-1_ that have been previously described in *P. multocida* [1,26], were not detected. In all isolates carrying AMR genes, these were organized within MDR-mediating ICEs (Figure 2, Figure 3 and Figure 4). In addition, isolates 1 and 6 harbored the macrolide resistance-mediating point mutation A2059C affecting all six copies of the 23S rRNA [42].

#### 2.3.1. Isolates Carrying a Tn*7407*-like ICE

Isolates 1, 3, 5, 6, and 7 harbored an ICE that was very closely related to ICE Tn*7407* (Table 1, Figure 2). ICE Tn*7407* was first described in a *P. multocida* isolate from a fatal case of BRD in a German Holstein-Friesian fattening calf [25]. The ICEs ranged in size from 69,567 to 78,401 bp, with identical 8-bp direct repeats (5′-ATTCAAAA-3′) present at their boundaries. As Tn*7407*-like elements, they were not integrated in a tRNA^Leu^ gene but appeared to have utilized the nucleotide sequence 5′-ATTCAAAA-3′ as the integration site [25]. Organized within two resistance regions (RR), RR1 and RR2, the ICEs harbored six (isolate 1) or seven (isolates 3, 5, 6, and 7) complete AMR genes. The ICE carried by isolate 1 matched Tn*7407* most closely, with a nucleotide sequence identity of 99.99% (Figure 2). Here, the tetracycline resistance gene *tet*(H) was part of RR1, while two copies of the sulfonamide resistance gene *sul2*, and single copies of the streptomycin resistance genes *strA* and *strB*, as well as the kanamycin/neomycin resistance gene *aphA1* were located within RR2. However, the ICEs harbored by isolates 6 and 7 differed from this ICE with a nucleotide sequence identity of 91.12%, but they showed 99.99% and 100% identity, respectively, to the sequence of a bovine *P. multocida* isolated in 2013 in the USA (GenBank accession no. CP015567) (Figure 2). Within RR1 of these ICEs, an additional 6782-bp segment was inserted between the insertion sequence (IS) element IS*Mha7* and the *tet*(H) gene, including open reading frames (ORFs) for a truncated *tet*(H), the spectinomycin/streptomycin resistance gene *aadA31*, as well as an IS*Pst2*-like element (Figure 2). Lastly, the ICEs detected in isolate 3 and 5 showed 88.73% nucleotide sequence identity to the ICE of isolate 1 and 97.38% nucleotide sequence identity to the ICEs carried by isolates 6 and 7 (Figure 2). Here, an additionally inserted IS*Mha7* element disrupted the gene *dnaB* coding for a replicative DNA helicase.

Furthermore, isolate 4 harbored an ICE that was disrupted into two parts with a segment of 732,885 bp between them. With a combined size of 78,401 bp, the two ICE parts were identical to the Tn*7407*-like ICEs of isolates 3 and 5, except for the right part of the ICE being present as reverse complement. Prior to the disruption, the ICE initially seemed to have also integrated into the nucleotide sequence 5′-ATTCAAAA-3′, with identical 8-bp repeats (5′-ATTCAAAA-3′) present at its boundaries. Since an additional IS*Mha7* element was found to have inserted into the ornithine decarboxylase gene *speF*, the observed disruption and inversion might be the result of a homologous recombination of the two IS*Mha7* elements in reverse orientation with inversion of the part between them (Figure 3). An IS*Mha7* element within *speF* was also detected in isolate 5 carrying the same ICE type and originating from the same farm; however, the ICE in isolate 5 was still complete. This might represent the state that existed in isolate 4 prior to the disruption and inversion, as displayed in Figure 3, thus supporting the formation theory of this observation. Accordingly, isolates 4 and 5 differed only in two SNPs, thus showing an even closer relationship than isolates 3 and 4 (13 SNPs, from the same animal), or 3 and 5 (11 SNPs), respectively (see Section 2.1, Table 2).

#### 2.3.2. Isolates Carrying a Tn*7406*-like ICE

Isolate 8 carried an ICE that showed a nucleotide sequence identity of 99.71% compared to ICE Tn*7406*, which has been first described in a *Mannheimia haemolytica* isolate that originated from the same fatal case of BRD as the first reported Tn*7407*-carrying *P. multocida* isolate mentioned under Section 2.3.1 [25] (Table 1, Figure 4). With 58,382 bp in size, the ICE from isolate 8 was 157 bp longer than Tn*7406*. It had not inserted into a chromosomal tRNA^Leu^ copy as Tn*7406* but into the non-coding nucleotide sequence 5′-GATTCAAAATC-3′ downstream of the chromosomal genes *hutZ* and *hutX* coding for a heme-degrading enzyme and an intracellular heme transport protein, respectively. Moreover, the ICE was flanked by 11-bp imperfect direct repeats (5′-GATTCAAAATC-3′ left terminus, 5′-GATTCGTGCTT-3′ right terminus). Although the integration site and right-hand terminus of the ICE in isolate 8 differed compared to Tn*7406*, the remaining parts of both ICEs, including the resistance regions, were identical (Figure 4). They comprised eight complete AMR genes, more precisely, the tetracycline resistance gene *tet*(Y), the streptomycin resistance genes *strA* and *strB*, the chloramphenicol resistance gene *catA3*, the phenicol resistance gene *floR*, the macrolide resistance genes *mef*(C) and *mph*(G), as well as the sulfonamide resistance gene *sul2*. An additional truncated *sul2* copy was inserted upstream of *catA3*.

## 3. Discussion

*P. multocida* as a common colonizer of the upper respiratory tract of mammals and birds has occasionally also been found to be associated with bovine mastitis [1,16]. Capsule structure and LPS composition are the basis for the classification of *P. multocida* into five capsular groups (A, B, D, E, and F) and 16 somatic serotypes (1–16) [45]. In this regard, certain capsular types are known to preferentially occur in connection with specific disorders [1,45]. In cattle, CAP type A and, to a lesser extent, type D are typically the causative agents of BRD, while CAP types B and E are commonly involved in hemorrhagic septicemia in Africa and Asia [1,45]. CAP type F may cause fatal peritonitis in calves [1,45,46]. The eight isolates investigated in this study all belonged to CAP type A and, except for one isolate, to LPS type 3. CAP:LPS type A:3 has been reported to be the most common serotype isolated from cases of BRD, with the isolates showing limited heterogeneity regarding outer membrane protein profiles and ribotyping [45]. Notably, a novel capsule biosynthetic locus allele, differing from *capA4* in a single mutation in the *hyaD* gene, was detected in isolates 3, 4, and 5 that originated from the same farm. Furthermore, the one isolate with the differing CAP:LPS type A:6 also showed another ST (ST7), while the remaining seven isolates belonged to ST1. The isolate and genome databases from PubMLST.org [47] allowed for a search for previously described *P. multocida* STs, which suggested that ST1 and ST7 are commonly detected in cattle within Europe among a few other countries. More precisely, ST1 has also been found in BRD-associated isolates from the United Kingdom and the Netherlands; a bovine mastitis isolate from the United Kingdom; bovine milk from Hungary; bovine nasal swabs from China and the USA; a bovine lung sample from Canada; and not further specified bovine samples from the USA, Canada, and China [47]. Moreover, ST7 has been reported from BRD-associated isolates from the United Kingdom and the Netherlands, bovine lung samples from the United Kingdom and the USA, as well as one bovine mastitis isolate from the United Kingdom [47]. Considering other or unspecified host species, ST1 seems to be even more widespread, since it has been detected in a turkey in the USA suffering from fowl cholera; a porcine pneumonia-associated isolate from China; unspecified porcine samples from South Korea and Australia; as well as in unknown hosts from Sri Lanka, Switzerland, and Morocco [47]. In contrast, German *P. multocida* isolates associated with hemorrhagic septicemia in cattle were found to belong to ST61 or ST64 [47]. With regard to the phylogenetic analysis, it was not surprising that isolates within our study belonging to the same ST and CAP:LPS type also showed closer relationships when comparing SNPs extracted from their core genome (Table 1 and Table 2). Moreover, the relatedness of the isolates mirrored the type of ICE they carried, either Tn*7406*-like or Tn*7407*-like. Overall, isolates 3, 4, and 5 from the same farm were most closely related.

The interpretation of MIC values and subsequent classification of the isolates as susceptible, intermediate, or resistant was difficult because clinical breakpoints for bovine *P. multocida* isolates from intramammary infections approved by the CLSI are not available [40]. Although the adopted breakpoints shown in Table A1 were suitable for orientation purposes within this study, it is questionable to what extent they can be used for treatment recommendations within the routine diagnosis of *P. multocida* mastitis isolates. Consequently, this underlines the need to establish further clinical breakpoints. In addition, it is worth mentioning that the breakpoints of erythromycin for human *Pasteurella* spp. [41] are considerably lower compared with the veterinary breakpoints of the other macrolides tested. This might lead to misinterpretations when erythromycin is tested as the only macrolide, of which no medicinal product for animals is currently approved in Germany [23]. Seven of the eight *P. multocida* isolates showed elevated MIC values of several antimicrobial classes that are relevant for mastitis therapy. Even more alarming, the respective resistance genes were all located on an ICE and thus might be transferred horizontally across bacterial strain, species, or even genus boundaries in a single event [1]. Moreover, the co-location of several AMR determinants on a single MGE increases the risk of co-selection processes, possibly resulting in the persistence of certain AMR traits in the bacterial population even in the absence of direct selection pressure [1]. In addition to the MDR-mediating ICEs, the macrolide resistance-mediating point mutation A2059C [35] was detected in all six 23S rRNA copies of the two isolates 1 and 6. The missing genotypic explanation for the β-lactam resistance observed in isolates 1 and 3 to 8 should be investigated further due to the great importance of these agents for mastitis therapy. Notably, an increase in MIC values did not always apply to all β-lactams tested in this study (Table 1). A possible mechanism that is worth investigating in a follow-up study might be a decreased ability or rate of entry of β-lactams into the bacterial cell [48]. Porins provide a path through the outer membrane of Gram-negative bacteria to small hydrophilic molecules, such as β-lactams, and numerous studies have already reported on acquired AMR due to loss or functional modification of porins in a large number of bacterial species [48].

*P. multocida* isolates harboring MDR-mediating ICEs, such as ICE*Pmu1* and ICE*Mh1*, have been detected in North America [30,49], while the novel ICE-*Pmu*ST394 has recently been described in *P. multocida* from Australia [26]. The first MDR-mediating ICE in *P. multocida* detected in Europe was Tn*7407* from a fatal case of BRD in a German calf [25]. MDR-mediating ICEs from *P. multocida* isolates associated with bovine mastitis have not been described prior to this study. These ICEs showed high similarity to ICE Tn*7407* (Figure 2 and Figure 3) and ICE Tn*7406* (Figure 4), respectively, the latter identified first in a *M*. *haemolytica* isolate from the same case of BRD as Tn*7407* [25]. Several novel ICEs with an almost identical core region compared to Tn*7406* but greatly differing resistance regions were recently also reported from BRD-associated *M*. *haemolytica* isolates from Germany [36]. Since *M*. *haemolytica* is a different bacterial species, the varying integration site of the Tn*7406*-like ICE in *P. multocida* isolate 8 from this study is not surprising. Genetic rearrangements resulting in the emergence of novel Tn*7406* variants as part of an ongoing evolution of ICEs among bovine *M*. *haemolytica* in Germany have already been discussed in a previous study [36]. Since here, a Tn*7406*-like ICE was also identified in a *P. multocida* isolate, horizontal cross-genus transfer of MDR-mediating ICEs seems to occur under in vivo conditions among different pathogens from cattle in Germany. Such a transfer has previously been confirmed under in vitro conditions [31]. Furthermore, the high similarities of the ICEs detected in this study to previously described ICEs from Germany and also the USA, emphasize the wide distribution of these MGEs among—to the best of our knowledge and with the exception of the three isolates from the same farm—epidemiologically unrelated *P. multocida* and *M*. *haemolytica* from cattle. Moreover, complete or truncated IS elements were frequently detected as part of the ICEs characterized in this study (Figure 2, Figure 3 and Figure 4). In particular, IS*Mha7* was involved in firstly described genetic modifications, such as the insertion of the element into the *dnaB* gene in isolates 3, 4, and 5, or its possible role in the disruption of the ICE in isolate 4 (Figure 2 and Figure 3). This underlines the relevance of these highly mobile IS elements for genetic rearrangement processes. These might be extensive—the inversion in isolate 4 affected about one third of the complete genome.

The molecular typing results and the similarities of the ICEs detected in the investigated *P. multocida* from bovine mastitis compared to those previously described in BRD-associated *Pasteurellaceae*, suggest the respiration tract as the origin of the mastitis isolates. This implication is in accordance with previous studies: On one hand, it was reported that the same *P. multocida* strain can cause respiratory disease as well as clinical mastitis in cattle [4]. On the other hand, there seems to be a limited degree of strain diversity among *P. multocida* associated with varying clinical symptoms including BRD and mastitis [50]. The infection might have manifested itself through lymphogenic or hematogenic spread [13], or via suckling calves/cows [16]. In four of the eight described isolates, cow and calf are not separated immediately after birth. Due to the rising public interest in animal welfare, the relevance of alternative housing systems with physical contact between cow and calf for a longer period of time increases [16]. Since the prevention of pathogen transmission is a major reason for the common early separation of cow and calf, this will be accompanied by additional challenges considering farm management and internal biosecurity to prevent the spread of pathogens within the livestock facility. Köllmann et al. showed that the transmission of certain mastitis-associated pathogens via suckling calves is very likely, resulting in an increase in intramammary infections in particular due to *P. multocida* [16]. Consequently, mastitis caused by uncommon pathogens, such as *P. multocida*, might be detected more frequently in alternative housing systems. AMR or even MDR of causative *P. multocida* isolates, as demonstrated by this study, can greatly limit the available treatment options for bovine mastitis. This emphasizes the importance of pathogen identification with subsequent AST for effective mastitis therapy. However, cows with mastitis due to *Pasteurella* spp. usually do not respond well to antimicrobial therapy [6,13], although it is not clear how big the role of potentially present AMR mechanisms in this is. Previous studies reported the combined administration of several antimicrobial classes to cows suffering from *P. multocida* mastitis [4,6], in one example, with the combined intramammary and parenteral application of six different antimicrobial agents to resolve the infections [4]. Wilson et al. showed that *Pasteurella* spp. had one of the highest somatic cell count numbers among 24 causative agents of bovine mastitis [7], which might be associated with more severe effects on udder health. In addition, affected animals might become life-threateningly ill when the pathogen’s endotoxins are present in the blood system [13]. MDR of *P. multocida*, as shown in our study, might render mastitis treatment even more difficult.

Finally, antimicrobial treatment is often indispensable in bovine mastitis therapy taking into account udder health, animal welfare, and economical aspects [19]. However, there is an urgent need for a reduction in antimicrobial usage in the dairy industry, since the dissemination of AMR poses a considerable threat to public health [19]. Currently, the most promising concepts include, in particular, the realization of evidence-based mastitis therapy approaches [19]. In this regard, the success depends on the implementation of standards for and monitoring of hygiene and animal welfare [19]. Furthermore, the quality of the microbiological analysis and of the available herd data, including somatic cell counts, milk yield, and previous mastitis cases, are of critical importance [19].

## 4. Materials and Methods

### 4.1. Bacterial Isolates

The eight *P. multocida* isolates included in this study were associated with clinical cases of bovine mastitis. They were detected in quarter milk samples sent to the Dairy Herd Consulting and Research Company (MBFG), a large mastitis diagnostic laboratory in Germany, during the years 2021 to 2023. In the Institute of Microbiology and Epizootics, School of Veterinary Medicine, Freie Universität Berlin, the isolates were confirmed as *P. multocida* by Matrix-Assisted Laser Desorption/Ionization Time of Flight Mass Spectrometry (MALDI-ToF MS). Furthermore, frozen stocks of Brain Heart Infusion bacterial liquid culture supplemented with 20% glycerol were prepared for longtime storage at −80 °C. The detailed characteristics and background information of the isolates can be found in Table 1.

### 4.2. Antimicrobial Susceptibility Testing (AST)

AST was performed according to the recommendations of the CLSI [40]. All isolates were subjected to broth microdilution using commercial microtiter plates (MICRONAUT-S Large Animal, MERLIN GmbH, Bornheim-Hersel, Germany; MICRONAUT-S, Small Animal, MERLIN GmbH, Bornheim-Hersel, Germany; Equine Plate Format, Sensititre™, Thermo Fisher Scientific, Waltham, MA, USA) as well as microtiter plates customized specifically for the German National Resistance Monitoring Program GE*RM*-Vet [51] (Sensititre™, Thermo Fisher Scientific, Waltham, MA, USA). In total, MIC values were obtained for 39 antimicrobial agents, including β-lactams (oxacillin, penicillin, ampicillin, amoxicillin/clavulanic acid 2:1, cefovecin, cephalexin, ceftiofur, cefquinome, cephalotin, cefotaxime, cefoperazone, cephazolin, ceftazidime, and imipenem), macrolides (clarithromycin, erythromycin, gamithromycin, tildipirosin, tilmicosin, and tulathromycin), a lincosamide (clindamycin), a pleuromutilin (tiamulin), (fluoro)quinolones (ciprofloxacin, enrofloxacin, marbofloxacin, pradofloxacin, and nalidixic acid), aminoglycosides (gentamicin, amikacin, neomycin, and streptomycin), tetracyclines (doxycycline, minocycline, and tetracycline), folate pathway inhibitors (trimethoprim/sulfamethoxazole 1:19), phenicols (florfenicol and chloramphenicol), an ansamycin (rifampicin), and a polymyxin (colistin).

Since CLSI-approved clinical breakpoints for *P. multocida* from bovine mastitis are not available, breakpoints for *P. multocida* from bovine respiratory tract infections, other animal species, or humans, respectively, were used for the interpretation of the obtained MIC values, as specified in Table A1. In addition, *P. multocida* isolates that showed high MIC values of oxacillin (≥4 mg/L), cephalexin (≥32 mg/L), cefquinome (≥32 mg/L), cephalotin (≥128 mg/L), cefotaxime (≥32 mg/L), cefoperazone (≥64 mg/L), ceftazidime (≥32 mg/L), clarithromycin (≥16 mg/L), neomycin (≥128 mg/L), and streptomycin (≥1024 mg/L) were considered as resistant despite the lack of CLSI-approved clinical breakpoints.

### 4.3. Whole-Genome Sequencing (WGS), Assembly, and Annotation

Genomic DNA was extracted, quality-checked, and visualized as described previously [36]. All samples were subjected to short-read and long-read sequencing. For short-read sequencing, 1 ng from the extracted DNA was used. Libraries were prepared with the Nextera^®^ XT DNA Library Preparation Kit (Illumina, Inc., San Diego, CA, USA) according to the manufacturer’s recommendations. The 2 × 300-bp paired-end sequencing in 40-fold multiplexes was performed on an Illumina MiSeq platform with the MiSeq reagent kit v3 (600-cycle; Illumina, Inc., San Diego, CA, USA). Using 200 ng of the same extracted DNA to generate a barcoded MinION one-dimensional library with the SQK-RBK114.24 kit (Oxford Nanopore Technologies, Oxford, UK), long-read sequencing for 48 h was performed with an Oxford Nanopore MinION device. Barcoded DNA was pooled and loaded onto a R10.4.1 flow cell (Oxford Nanopore Technologies, Oxford, UK) to carry out multiplexed sequencing.

For Illumina short-reads, Trim Galore v0.6.10 (RRID: SCR_011847) and FastQC v0.12.1 [52] were used for adapter trimming and quality checks, respectively. Nanopore data sets were base-called and demultiplexed into quality-tagged sequence reads with 4000 reads per fastq-file with Dorado v7.3.11 integrated in the MinKnow^TM^ software v24.02.8 (Oxford Nanopore Technologies, Oxford, UK). Here, Porechop v0.2.4 [53] and Filtlong v0.2.1 [54] were used for adapter trimming and elimination of reads below 1000 bp or sequence quality worse than QV 7.5. LongQC [55] was used for the quality checks. In the following, Unicycler v0.4.9 [56] and the Flye algorithm in MaSuRCA v4.1.0 [57] were used for a hybrid assembly of MinION long-reads and Illumina short-reads to generate closed genomes. An additional third assembly was performed with Flye v2.9.2 [58] using the MinION long-reads, which were polished with NextPolish v1.4.1 [59] using Illumina short-reads. The three resulting closed, complete genomes were then used to generate a corrected consensus sequence with Geneious v11.1.5 (Biomatters, Ltd., Auckland, New Zealand), which was annotated with Prokka v1.14.5 [60] and Bakta v1.9.4 [61]. Finally, the nucleotide sequences were deposited at GenBank (https://www.ncbi.nlm.nih.gov/genbank/ (accessed on 1 February 2025)) within BioProject PRJNA1176323 under the accession numbers CP172106 (1), CP172105 (2), CP172104 (3), CP172103 (4), CP172102 (5), CP172101 (6), CP172100 (7), and CP172107 (8).

### 4.4. Molecular Typing, Investigation of AMR Genotype and ICEs, and Phylogenetic Analysis

Further analysis of the whole-genome sequences and verification of automatic annotations were carried out using Geneious v11.1.5 (Biomatters Ltd., Auckland, New Zealand) and the Basic Local Alignment Search Tool v2.16.0 [62]. MLST (multi-host scheme) [47], CAP, and LPS types [38] were deduced from the sequences. AMR genes were identified using ABRicate [63] with the NCBI AMRFinderPlus [64] and ResFinder [65] databases. Mobile elements were detected with the ISfinder tool [66,67]. The novel ICE as well as AMR-mediating mutational changes were identified using MAFFT alignment in Geneious v11.1.5 (Biomatters Ltd., Auckland, New Zealand). For the phylogenetic analysis, a core-genome of all *P. multocida* isolates was generated using the bacterial pangenome analysis pipeline Panaroo [44] with default settings. SNPs were extracted from the filtered core-genome alignments from Panaroo with SNP-sites [43].

## 5. Conclusions

AMR is a global public health problem, which also affects the bacterial pathogens involved in bovine mastitis. In this study, seven of eight *P. multocida* isolates from clinical cases of bovine mastitis in Germany carried MDR-mediating ICEs. Molecular typing of the isolates and comparison of the detected ICEs with those previously described in *P. multocida* and *M. haemolytica* from respiratory tract infections of cattle suggest that the respiratory tract may be the reservoir of the investigated *P. multocida* from bovine mastitis. ICEs are self-transmissible elements that can move between bacteria of the same but also other bacterial species and genera. Due to potential horizontal gene transfer events and co-selection processes, the presence of these ICEs might enhance the spread of AMR genes and, thus, diminish treatment options. Monitoring of such ICEs is warranted to follow their dissemination, while elimination of the respective isolates is difficult as *P. multocida* often occurs as a commensal in the oropharynx of cattle. The only option to control MDR-positive *P. multocida* and to get rid of the aforementioned MDR-mediating ICEs is to use antimicrobial agents for mastitis therapy for which no antimicrobial resistance genes are located on these ICEs. Even though *P. multocida* is not a major mastitis pathogen, the presence of MDR-mediating ICEs in this bacterial species strongly suggests that pathogen identification, followed by AST, is essential prior to the start of antimicrobial therapy.

## Figures and Tables

**Table 1:** Characteristics and AMR profiles of the eight *P. multocida* isolates.

ID	Year	Federal State	CAP:LPS	MLST	ICE (Size)	AMR Phenotype ^b^	AMR Genotype
1	2021	North Rhine-Westphalia	A:3	1	Tn*7407*-like (69,567 bp)	PEN, AMP, AUG, XNL, CEQ, CEP, FOT, FOP, FAZ, CLA, ERY, GAM, TDP, TIL, TUL, NEO, STR, TET, SXT	*strA*, *strB*, *aphA1*, *tet*(H), *sul2* (2 x) ^c^
2	2022	Lower Saxony	A:6	7	-	-	-
3 **^a^**	2022	North Rhine-Westphalia	A:3	1	Tn*7407*-like (78,401 bp)	OXA, PEN, AMP, AUG, CFV, CEX, XNL, CEQ, CEP, FOT, FOP, FAZ, NEO, STR, TET, SXT	*strA*, *strB*, *aphA1*, *aadA31*, *tet*(H), *sul2* (2 x)
4 ^a^	2022	North Rhine-Westphalia	A:3	1	Tn*7407*-like (disrupted, 78,401 bp)	PEN, AMP, AUG, CFV, CEX, XNL, FAZ, TAZ, NEO, STR, TET, SXT	*strA*, *strB*, *aphA1*, *aadA31*, *tet*(H), *sul2* (2 x)
5 ^a^	2022	North Rhine-Westphalia	A:3	1	Tn*7407*-like (78,401 bp)	OXA, PEN, AMP, AUG, CFV, CEX, XNL, CEQ, CEP, FOT, FOP, NEO, STR, TET, SXT	*strA*, *strB*, *aphA1*, *aadA31*, *tet*(H), *sul2* (2 x)
6	2023	Lower Saxony	A:3	1	Tn*7407*-like (76,349 bp)	PEN, AMP, AUG, XNL, CEQ, CEP, FOT, FOP, CLA, ERY, GAM, TDP, TIL, TUL, NEO, STR, TET, SXT	*strA*, *strB*, *aphA1*, *aadA31*, *tet*(H), *sul2* (2 x) ^c^
7	2023	Lower Saxony	A:3	1	Tn*7407*-like (76,349 bp)	OXA, PEN, AMP, AUG, CFV, CEX, XNL, CEQ, CEP, FOT, NEO, STR, TET, SXT	*strA*, *strB*, *aphA1*, *aadA31*, *tet*(H), *sul2* (2 x)
8	2023	Saxony-Anhalt	A:3	1	Tn*7406*-like (58,382 bp)	CLA, ERY, GAM, TUL, STR, TET, SXT, FFN, CHL	*mef*(C), *mph*(G), *strA*, *strB*, *tet*(Y), *sul2*, *floR*, *catA3*

CAP:LPS, capsular:lipopolysaccharide type; MLST, multi-locus sequence type; OXA, oxacillin, PEN, penicillin, AMP, ampicillin; AUG, amoxicillin/clavulanic acid (2:1); CFV, cefovecin; CEX, cephalexin; XNL, ceftiofur; CEQ, cefquinome; CEP, cephalotin; FOT, cefotaxime; FOP, cefoperazone; FAZ, cephazolin; TAZ, ceftazidime; CLA, clarithromycin; ERY, erythromycin; GAM, gamithromycin; TDP, tildipirosin; TIL, tilmicosin; TUL, tulathromycin; NEO, neomycin; STR, streptomycin; TET, tetracycline; SXT, trimethoprim/sulfamethoxazole (1:19); FFN, florfenicol; CHL, chloramphenicol. **^a^** These isolates originated from the same farm; isolates 3 and 4 were obtained from different udder quarters of the same animal. ^b^ Since CLSI-approved clinical breakpoints for *P. multocida* from intramammary infections are not available, breakpoints for *P. multocida* from bovine respiratory tract infections, other animal species, or humans, respectively, were applied (Table A1, Appendix A) [40,41]. ^c^ These isolates harbored the macrolide resistance-mediating point mutation A2059C affecting all six copies of the 23S rRNA [42].

**Table 2:** SNP distance matrix of the eight *P. multocida* isolates created with SNP-sites [43] using the core genome generated with Panaroo [44].

Isolate ID	1	2	3	4	5	6	7	8
**1**	0	13,872	41	32	30	18	30	174
**2**	13,872	0	13,878	13,869	13,867	13,874	13,869	13,846
**3**	41	13,878	0	13	11	43	37	186
**4**	32	13,869	13	0	2	34	28	177
**5**	30	13,867	11	2	0	32	26	175
**6**	18	13,874	43	34	32	0	32	177
**7**	30	13,869	37	28	26	32	0	175
**8**	174	13,846	186	177	175	177	175	0

## Data Availability

All data presented in this study are available in the text, figures, and tables of the main article. Whole-genome sequences of the investigated *P. multocida* isolates are available at GenBank within BioProject PRJNA1176323 under the accession numbers CP172106 (1), CP172105 (2), CP172104 (3), CP172103 (4), CP172102 (5), CP172101 (6), CP172100 (7), and CP172107 (8) (https://www.ncbi.nlm.nih.gov/genbank/ (accessed on 1 February 2025)).

## Abbreviations

The following abbreviations are used in this manuscript:

BRD	Bovine respiratory disease
USA	United States of America
AMR	Antimicrobial resistance
ICE	Integrative and conjugative element
MGE	Mobile genetic element
MDR	Multidrug resistance
MIC	Minimal inhibitory concentration
WGS	Whole-genome sequencing
AST	Antimicrobial susceptibility testing
ST	Sequence type
CAP	Capsular
LPS	Lipopolysaccharide
MLST	Multi-locus sequence type
OXA	Oxacillin
PEN	Penicillin
AMP	Ampicillin
AUG	Amoxicillin/clavulanic acid (2:1)
CFV	Cefovecin
CEX	Cephalexin
XNL	Ceftiofur
CEQ	Cefquinome
CEP	Cephalotin
FOT	Cefotaxime
FOP	Cefoperazone
FAZ	Cephazolin
TAZ	Ceftazidime
CLA	Clarithromycin
ERY	Erythromycin
GAM	Gamithromycin
TDP	Tildipirosin
TIL	Tilmicosin
TUL	Tulathromycin
NEO	Neomycin
STR	Streptomycin
TET	Tetracycline
SXT	Trimethoprim/sulfamethoxazole (1:19)
FFN	Florfenicol
CHL	Chloramphenicol
SNP	Single-nucleotide polymorphism
CLSI	Clinical and Laboratory Standards Institute
RR	Resistance region
IS	Insertion sequence
MALDI-ToF MS	Matrix-Assisted Laser Desorption/Ionization Time of Flight Mass Spectrometry

## Appendix A

**Table A1:** Clinical breakpoints adopted for the MIC value interpretation of the *P. multocida* isolates from bovine mastitis.

Antimicrobial Agent	Host Species/ Body Site ^1^	Interpretive Categories and MIC Breakpoints in mg/L ^2^			Reference
		S	I	R	
Oxacillin	NA	-	-	-	-
Penicillin	Cattle, RT	≤0.25	0.5	≥1	[40]
Ampicillin	Cattle, RT	≤0.03	0.06–0.12	≥0.25	[40]
Amoxicillin/ Clavulanic acid 2:1	Cats, SST, UT	≤0.25/0.12	0.5/0.25	≥1/0.5	[40]
Cefovecin	Cats, SST	≤0.12	0.25	≥0.5	[40]
Cephalexin	NA	-	-	-	-
Ceftiofur	Cattle, RT	≤2	4	≥8	[40]
Cefquinome	NA	-	-	-	-
Cephalotin	NA	-	-	-	-
Cefotaxime	NA	-	-	-	-
Cefoperazone	NA	-	-	-	-
Cephazolin	Dogs, RT, SST	≤2	4	≥8	[40]
Ceftazidime	NA	-	-	-	-
Imipenem	NA	-	-	-	-
Clarithromycin	NA	-	-	-	-
Erythromycin	Humans	≤0.5	1	≥2	[41]
Gamithromycin	Cattle, RT	≤4	8	≥16	[40]
Tildipirosin	Cattle, RT	≤8	16	≥32	[40]
Tilmicosin	Swine, RT	≤16	-	≥32	[40]
Tulathromycin	Cattle, RT	≤16	32	≥64	[40]
Clindamycin	NA	-	-	-	-
Tiamulin	NA	-	-	-	-
Ciprofloxacin	NA	-	-	-	-
Enrofloxacin	Cattle, RT	≤0.25	0.5–1	≥2	[40]
Marbofloxacin	NA	-	-	-	-
Pradofloxacin	Cattle, RT	≤0.12	0.25	≥0.5	[40]
Nalidixic acid	NA	-	-	-	-
Gentamicin	NA	-	-	-	-
Amikacin	NA	-	-	-	-
Neomycin	NA	-	-	-	-
Streptomycin	NA	-	-	-	-
Doxycycline	Humans	≤0.5	-	-	[41]
Minocycline	NA	-	-	-	-
Tetracycline	Cattle, RT	≤2	4	≥8	[40]
Trimethoprim/ Sulfamethoxazole 1:19	Humans	≤0.5/9.5	-	-	[41]
Florfenicol	Cattle, RT	≤2	4	≥8	[40]
Chloramphenicol	Humans	≤2	-	-	[41]
Rifampicin	NA	-	-	-	-
Colistin	NA	-	-	-	-

^1^ NA, no clinical breakpoint available in any of the referenced documents; RT, respiratory tract; SST, skin and soft tissue; UT, (lower) urinary tract. ^2^ S, susceptible; I, intermediate; R, resistant.
